# Dynamic Changes in Endothelial Cell Adhesion Molecule Nepmucin/CD300LG Expression under Physiological and Pathological Conditions

**DOI:** 10.1371/journal.pone.0083681

**Published:** 2013-12-23

**Authors:** Eiji Umemoto, Akira Takeda, Soojung Jin, Zhijuan Luo, Naoki Nakahogi, Haruko Hayasaka, Chun Man Lee, Toshiyuki Tanaka, Masayuki Miyasaka

**Affiliations:** 1 Laboratory of Immunodynamics, Department of Microbiology and Immunology, Osaka University Graduate School of Medicine, Suita, Osaka, Japan; 2 Laboratory of Immunodynamics, World Premier International Research Center Initiative-Immunology Frontier Research Center, Osaka University, Suita, Osaka, Japan; 3 Medical Center for Translational Research, Osaka University Hospital, Suita, Osaka, Japan; 4 Laboratory of Immunobiology, School of Pharmacy, Hyogo University of Health Sciences, Kobe, Japan; Okayama University, Japan

## Abstract

Vascular endothelial cells often change their phenotype to adapt to their local microenvironment. Here we report that the vascular endothelial adhesion molecule nepmucin/CD300LG, which is implicated in lymphocyte binding and transmigration, shows unique expression patterns in the microvascular endothelial cells of different tissues. Under physiological conditions, nepmucin/CD300LG was constitutively and selectively expressed at the luminal surface of the small arterioles, venules, and capillaries of most tissues, but it was only weakly expressed in the microvessels of the splenic red pulp and thymic medulla. Furthermore, it was barely detectable in immunologically privileged sites such as the brain, testis, and uterus. The nepmucin/CD300LG expression rapidly decreased in lymph nodes receiving acute inflammatory signals, and this loss was mediated at least in part by TNF-α. It was also down-regulated in tumors and tumor-draining lymph nodes, indicating that nepmucin/CD300LG expression is negatively regulated by locally produced signals under these circumstances. In contrast, nepmucin/CD300LG was induced in the high endothelial venule-like blood vessels of chronically inflamed pancreatic islets in an animal model of non-obese diabetes. Interestingly, the activated CD4^+^ T cells infiltrating the inflamed pancreas expressed high levels of the nepmucin/CD300LG ligand(s), supporting the idea that nepmucin/CD300LG and its ligand interactions are locally involved in pathological T cell trafficking. Taken together, these observations indicate that the nepmucin/CD300LG expression in microvascular endothelial cells is influenced by factor(s) that are locally produced in tissues, and that its expression is closely correlated with the level of leukocyte infiltration in certain tissues.

## Introduction

Endothelial cells (ECs) form the inner lining of all blood vessels, from the large conduit vessels (arteries and veins) to the small ones (arterioles, venules, and capillaries). While ubiquitous in the body, microvascular ECs display heterogeneity in their morphology, structure, and function, depending on their location [1]–[3]. For example, the brain microvasculature is lined by a continuous EC layer with well-developed tight junctions, serving as the blood brain barrier, while the spleen and bone marrow sinusoids are formed by discontinuous ECs that readily allow cellular trafficking. The heterogeneity of microvessels has been delineated by gene and protein expression patterns [4], [5]. Some vascular markers, such as CD31/PECAM-1, are widely distributed in a variety of tissues [6], whereas PV-1/PAL-E/PLVAP, the molecule recognized by the MECA-32 mAb [7], is expressed in medium-sized vessels in the lung, intestinal villi, and dermis, but not in the brain, heart, or kidney glomeruli [8], [9].

High endothelial venules are highly specialized blood vessels found in secondary lymphoid organs, such as lymph nodes (LNs) and Peyer’s patches, and they selectively mediate a constitutive migration of lymphocytes from the blood to the LN parenchyma [10], [11]. The interactions of blood-borne lymphocytes and high endothelial venules consist of a multistep adhesion cascade: lymphocyte rolling, chemokine-induced firm adhesion, and transendothelial migration across the ECs. In particular, lymphocyte rolling on the luminal surface of LN high endothelial venules is regulated by the interaction of lymphocyte L-selectin and specific carbohydrate determinants presented by endothelial sialomucins [12], [13]. These carbohydrate-based L-selectin ligands are recognized by the MECA-79 mAb, and the complex of reactive glycoproteins is collectively called the peripheral node addressin (PNAd) [14].

Microvascular ECs show specific characteristics under pathological conditions. The endothelial lining of tumor blood vessels usually arises from the proliferation of neighboring normal ECs, but it acquires abnormal properties upon interacting with the tumor’s extracellular matrices and exposure to tumor-derived mediators, such as growth factors. Tumor vessels frequently exhibit excessive branching, uneven diameters, uncontrolled permeability, and increased fenestrations [1], [15]. They also express various levels of adhesion molecules for circulating leukocytes; for example, VEGF stimulation is reported to increase the expressions of ICAM-1 and VCAM-1 on tumor ECs [16], and E-selectin expression is high in proliferating ECs in tumors [17].

During chronic inflammation, blood vessels expressing PNAd often appear at inflamed sites. In humans, this has been documented in rheumatoid arthritis, thyroiditis, psoriasis, Crohn’s disease, ulcerative colitis, and the gastritis associated with *Helicobacter pylori* infection [12], [18]. In experimental animals such as non-obese diabetic (NOD) mice, which develop diabetes as a result of leukocyte infiltration into pancreatic islets, high endothelial venule-like blood vessels expressing PNAd [19], [20] with L-selectin ligand activity appear in the inflamed islets [19]. Thus, ECs appear to be “plastic,” that is, able to adapt to their microenvironment in the vascular tree. However, the heterogeneity of vascular ECs observed under physiological and pathological conditions remains poorly understood.

Nepmucin/CD300LG is a type I membrane protein possessing a mucin-like domain and a single V-type Ig domain [13], [21]. It is also referred to as CLM-9 [22]. Nepmucin/CD300LG is expressed in the vascular ECs of certain tissues, including the high endothelial venules in LNs but not in Peyer’s patches, and plays multiple roles in lymphocyte migration across ECs; high endothelial venule-associated nepmucin/CD300LG mediates the L-selectin-dependent lymphocyte rolling upon the appropriate oligosaccharide modification of its mucin-like domain [21]. Nepmucin/CD300LG mediates heterotypic and homotypic cell adhesion via its Ig domain. Endothelial nepmucin/CD300LG promotes lymphocyte binding through interactions with unknown ligand(s) on lymphocytes [21], and mediates lymphocyte transendothelial migration via homotypic interactions between adjacent ECs, at least *in vitro* [23]. In addition, a recent genome-wide association study (GWAS) reported that a polymorphism observed in *nepmucin/CD300LG* gene is associated with serum HDL-cholesterol levels [24].

In this study, we showed that nepmucin/CD300LG was selectively expressed in microvessels, including small arterioles, venules, and capillaries in various tissues. In immune-related organs, nepmucin/CD300LG was particularly expressed in the splenic white pulp, thymic cortex, and LNs, but only weakly in the splenic red pulp or thymic medulla. By contrast, its expression was very weak or absent in immunologically privileged sites. The nepmucin/CD300LG expression was rapidly down-regulated by inflammatory signals or tumor-derived factors, and it was induced in the chronically inflamed pancreatic islets of NOD mice. Interestingly, nepmucin/CD300LG bound to a significantly greater proportion of CD4^+^ T cells in pancreatic islets than in the LNs or spleen, supporting the idea that nepmucin/CD300LG has a functional role in chronic lymphocyte infiltration in the pancreas.

## Materials and Methods

### Animals

C57BL/6 and BALB/c mice were purchased from Japan SLC, and used to examine nepmucin expression in normal and acutely inflamed tissues. C3H/HeN and SCID mice (C.B-17/Icr-scid/scid) were from Japan SLC and Japan Clea, respectively, and used for the analysis of nepmucin expression in tumor-associated tissues. NOD mice (Japan Clea) were used as an animal model for type I diabetes. Normal ICR mice (Japan Clea) were used as a negative control for NOD studies. Mice were housed under specific pathogen-free conditions. All animal experiments were performed under an experimental protocol approved by the Ethics Review Committee for Animal Experimentation of the Osaka University Graduate School of Medicine.

### Reagents and antibodies

Monoclonal antibodies (mAbs) against mouse nepmucin/CD300LG (ZAQ2 and ZAQ5), and chimeric proteins in which the extracellular domain of nepmucin was fused with the Fc portion of human IgG were prepared as described previously [21]. FITC-conjugated anti-CD31 mAb (MEC13.3) was purchased from BD Pharmingen. Anti-PV-1 mAb (MECA-32) was from the Developmental Studies Hybridoma Bank (the University of Iowa). Anti-PNAd (MECA-79) and anti-MAdCAM-1(MECA-367) mAbs were purified using a size-exclusion column (Toyopearl TSK HW55, Tosoh) and a HiTrap protein G column (GE Healthcare), respectively. Cy3-conjugated anti-α-smooth muscle actin mAb (1A4) and complete Freund’s adjuvant (CFA) were from Sigma. Recombinant mouse TNF-α was from R&D Systems.

### Whole-mount microscopic analysis

Mice were intravenously injected with Alexa Fluor 594- or 647-conjugated anti-nepmucin mAb. Fifteen minutes after the injection, the mice were euthanized, transcardially perfused with PBS, and fixed with 4% paraformaldehyde (PFA) in saline. For α-smooth muscle actin staining, collected tissues were further fixed with 4% PFA and permeabilized with 0.1% saponin for 1 hour. After non-specific binding was blocked with 5 µg/ml mouse γ-globulin and 5% fetal calf serum (FCS), the tissues were incubated with Cy3-conjugated anti-α-smooth muscle actin for 2 hours, followed by treatment with increasing concentrations (10%, 20%, and 30%) of sucrose. The immunofluorescent signals were observed with an FV1000-D confocal laser-scanning microscope (Olympus).

### Immunohistochemistry

Nepmucin expression was evaluated at the protein level by immunohistological analysis using mAb specific to nepmucin/CD300LG. For fluorescence immunohistochemistry, frozen sections of the thymus, liver, spleen, and kidney were fixed in methanol, and blocked with PBS containing 10% FCS and 20 µg/ml rat IgG. The sections were then incubated with Alexa Fluor 594-conjugated anti-nepmucin mAb (ZAQ5) and Alexa Fluor 647-conjugated anti-PV-1 mAb. For the small intestine, frozen sections were blocked with PBS containing 10% FCS, 20 µg/ml mouse γ-globulin, and 20 µg/ml goat IgG, and then incubated with an anti-nepmucin mAb or control rat IgG (Cappel), followed by Cy3-conjugated F(ab’)_2_ anti-rat IgG (Jackson ImmunoResearch). After being blocked with rat IgG, the sections were further incubated with Alexa Fluor 647-conjugated anti-PV-1 mAb. For the aorta/vena cava and LNs, frozen sections were co-incubated with Alexa Fluor 594-conjugated anti-nepmucin mAb (ZAQ5) and FITC-conjugated anti-CD31 mAbs.

For enzyme-based immunohistochemistry, frozen sections were fixed, blocked with FCS and goat IgG, and incubated with anti-nepmucin mAb (ZAQ2 or ZAQ5), anti-CD31 mAb, or rat IgG. After 0.3% H_2_O_2_ treatment, the samples were incubated with horseradish peroxide-conjugated goat anti-rat IgG, and the signal was developed with the Metal Enhanced DAB substrate kit (Thermo Scientific).

### Preparation of stromal cells

Stromal cells that include ECs were prepared from LNs as described previously [25].The cells (6.0×10^5^) were then seeded on a 48-well plate precoated with rat collagen type I (Millipore), and cultured in 20% FCS-containing Dulbecco’s modified Eagle medium. Two hours later, non-adherent cells were gently removed, and adherent cells were collected immediately (time 0) or after a 5-hours additional cell culture.

### Quantitative PCR

The expression level of *nepmucin* gene was evaluated by quantitative PCR. Total RNA was isolated using TRIzol reagent (Life Technologies) from mouse tissues or GenElute Mammalian total RNA Miniprep kit (Sigma-Aldrich) from freshly-prepared cells, and single-strand cDNA was synthesized using M-MLV reverse transcriptase (Promega) with a random primer. PCR was performed using the GoTaq qPCR system (Promega) at 95°C for 10 minutes followed by 40 cycles at 95°C for 15 seconds and 60°C for 1 minute. The PCR primer pairs were nepmucin: 5′-ACCTCAACCCTTCTGCTGTG-3′ (forward) and 5′-ATCACGTCCTCCTTGTCGTC-3′ (reverse), CD31: 5′-CCGATTCCTGGATTGCGAGA-3′ (forward) and 5′-AGCGCCTCTGAGTCTCTGTA-3′ (reverse), ICAM-1: 5′-CACGTGCTGTATGGTCCTCG-3′ (forward) and 5′-TAGGAGATGGGTTCCCCCAG-3′ (reverse), DARC: 5′-GAGCCACAGCTGCTGACAGAG-3′ (forward) and 5′-GCAGCTGGTGTCAGGCTGTAG-3′ (reverse), and GAPDH: 5′-CCTCGTCCCGTAGACAAAATG-3′ (forward) and 5′-TCTCCACTTTGCCACTGCAA-3′ (reverse).

### Injection of tumor cells

The human pancreatic carcinoma cell line MIA PaCa-2 was obtained from the Cell Resource Center for Biomedical Research, Institute of Development, Aging and Cancer, Tohoku University (Sendai, Japan). MIA PaCa-2 cells (1.2×10^7^ cells) were subcutaneously injected into the flank of SCID mice that had been treated with anti-IL-2Rβ mAb (TM-β1, 300 µg/mouse) [26]. Eighteen days later, the tumor tissues were collected. A derivative of the LM8 murine osteosarcoma cell line, LM8G5, which has a high potential for metastasis to the liver, was previously established in our laboratory [27]. LM8G5 cells (1×10^6^ cells) were intravenously injected into the ileocolic vein of C3H/HeN mice, and the liver was harvested 7 days later. B16-F10 mouse melanoma cells (1×10^5^ cells) were subcutaneously inoculated into the flank of C57BL/6 mice, and the draining axillary LNs were collected at the indicated time points.

### Flow cytometric analysis

Pancreases were digested with 400 Mandel U/ml collagenase D (Roche) and 10 µg/ml DNase I (Roche) in RPMI 1640 containing 10% FCS while being stirred continuously at 37°C for 60 min. Lymphocyte-enriched samples were then obtained by centrifugation on a discontinuous 40/80% Percoll gradient. The cells were incubated with mouse γ-globulin, and the mixture of nepmucin-chimeric proteins and PE-conjugated F(ab’)_2_ anti-human IgG [28] in Dulbecco’s modified Eagle medium, followed by APC-conjugated anti-CD4 mAb. Dead cells were excluded using 7-Amino-actinomycin D (7-AAD). The cells were analyzed by a FACSCanto II (BD Biosciences).

### Statistical analysis

A Student’s *t* test was applied to compare the statistical difference between two conditions.

## Results

### Nepmucin/CD300LG is selectively expressed in microvascular ECs in various tissues

**Figure 1:**
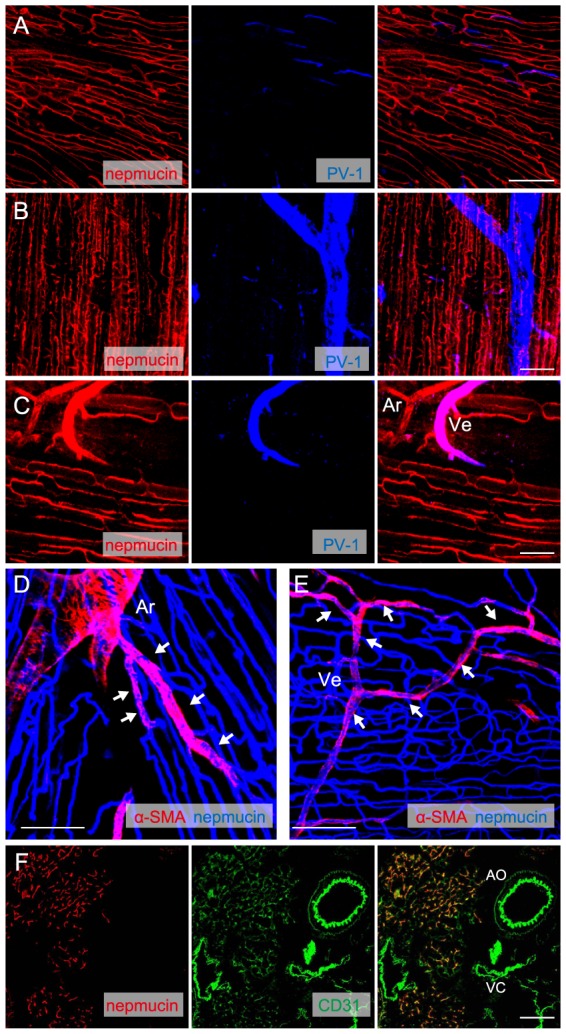
Nepmucin/CD300LG is selectively expressed in microvessels. (A-C) Whole-mount images of vasculature in the uvea (A), diaphragm (B), and trachea (C). Mice were intravenously injected with Alexa Fluor 594-conjugated anti-nepmucin mAb (red) and Alexa Fluor 647-conjugated anti-PV-1 mAb (blue), and transcardially perfused with 4% PFA. Collected tissues were observed using a laser-scanning confocal microscope, and Z-series images of each tissue were projected into one plane to show the vascular structures. (D, E) Mice were intravenously injected with Alexa Fluor 647-conjugated anti-nepmucin mAb (blue). After fixation, collected tissues were stained with Cy3-conjugated anti-α-smooth muscle actin mAb (red). The arterioles (D) and venules (E) were identified by the wrapping morphology of α-smooth muscle actin-expressing perivascular cells. Nepmucin-expressing vascular fragments enwrapped by α-smooth muscle actin^+^ cells are indicated by arrows. (F) A frozen section of tissue containing the ventral aorta and inferior vena cava was stained with Alexa Fluor 594-conjugated anti-nepmucin mAb (red) and FITC-conjugated anti-CD31 mAb (green). Note that nepmucin staining was absent in the aorta (AO) and vena cava (VC), whereas it was detectable in the capillaries of the surrounding adipose tissues. Ar: arteriole, Ve: venule. Scale bars, 100 µm.

To examine the constitutive expression of nepmucin in the vasculature, we first injected a mixture of Alexa Fluor 594-conjugated anti-nepmucin mAb with Alexa Fluor 647-conjugated anti-PV-1 mAb (an EC marker) intravenously into mice, and performed a whole-mount analysis of various tissues by confocal microscopy. As shown in Figure 1A-C, nepmucin was highly expressed along the luminal region of capillaries, arterioles, and venules, which all have relatively small diameters, but not of medium-sized PV-1^+^ vessels in the uvea, diaphragm, or trachea. To confirm the nepmucin expression in arterioles and venules, we stained the trachea with anti-nepmucin and anti-α-smooth muscle actin mAbs, because α-smooth muscle actin expression is confined to the perivascular cells of arterioles and venules, which can be identified by their wrapping morphology [29]. As shown in Figure 1D and E, nepmucin was readily detectable in the small arterioles and venules enwrapped by α-smooth muscle actin^+^ perivascular cells, but was undetectable in the large arterioles/venules. Conventional immunohistological analyses revealed the absence of nepmucin expression in the aorta and vena cava (Figure 1F). These data indicated that nepmucin preferentially marks microvascular ECs, including those of capillaries, small arterioles, and venules, and not those of larger blood vessels.

### Nepmucin/CD300LG shows heterogeneous expression in lymphoid organs, and is undetectable in immunologically privileged sites

**Figure 2:**
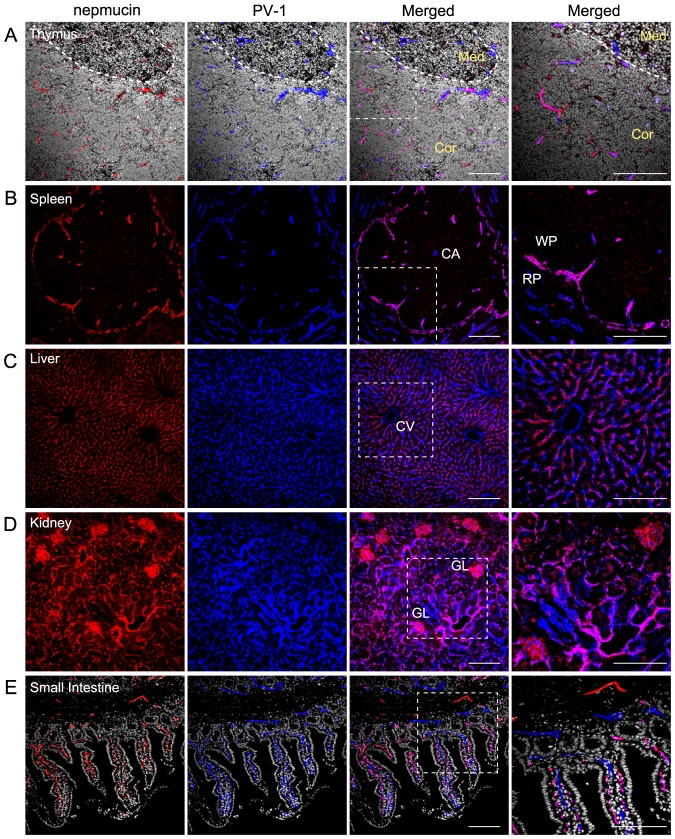
Nepmucin/CD300LG shows heterogeneous expression patterns in distinct compartments of the thymus and spleen. (A-E) Frozen sections of thymus (A), spleen (B), liver (C), kidney (D), and small intestine (E) were stained with an anti-nepmucin mAb (Alexa Fluor 594; red) and anti-PV-1 mAb (Alexa Fluor 647; blue). Cryosections of the thymus (A) and small intestine (E) were further incubated with Hoechst 33342 (white). In the thymus (A), the cortico-medullary junction is indicated by a dotted line. Med: medulla, Cor: Cortex, CA: central artery, WP: white pulp, RP: red pulp, CV: central vein, GL: glomerulus. Scale Bars, 100 µm.

Because nepmucin was initially identified in the high endothelial venules of LNs [21], we examined its expression in other lymphoid organs. To this end, we performed immunohistochemistry for nepmucin along with PV-1. In the thymus, strong nepmucin expression was observed in the small blood vessels of the cortex, but it was absent from the medullary blood vessels, whereas PV-1 was broadly expressed in all the blood vessels (Figure 2A). In the spleen, nepmucin was predominantly expressed in the marginal sinus and in some small blood vessels of the white pulp, but was undetectable in the central arteriole. In the red pulp, the nepmucin expression was limited, in sharp contrast to the broad expression of PV-1 (Figure 2B). Thus, nepmucin was preferentially expressed in distinct compartments of these immunological organs.

Unlike the above-mentioned lymphoid tissues, nepmucin was broadly expressed in the microvessels of the liver, kidney, and intestine. In the liver, the nepmucin signal widely overlapped with that of PV-1 in the sinusoidal ECs, while it was absent from the central vein and portal vein (Figure 2C). In the kidney, nepmucin was expressed in the glomerular and interstitial capillaries (Figure 2D). In the small intestine, nepmucin was strongly expressed in the capillaries of the lamina propria, whereas PV-1 was detectable in both the capillaries and lymphatics. Nepmucin was also expressed in the venules of the muscle layer, where PV-1 was undetectable (Figure 2E).

**Figure 3:**
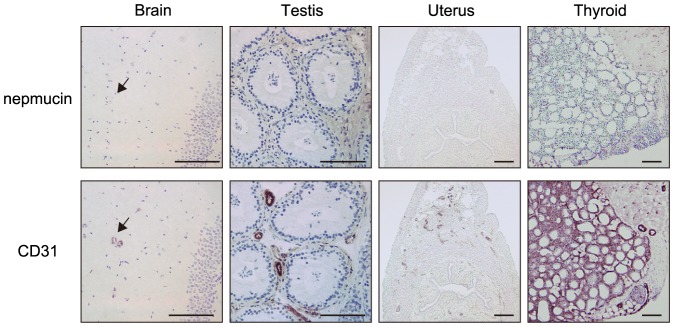
Nepmucin/CD300LG expression is apparently absent from immunologically privileged sites. Frozen sections of the indicated tissues were incubated with an anti-nepmucin mAb or anti-CD31 mAb, followed by HRP-conjugated anti-rat IgG. The reaction was then developed with DAB substrate. Arrows indicate blood vessels in the brain. No nepmucin expression was observed in these tissues. Scale bars, 100 µm.

In stark contrast, nepmucin expression was uniformly absent from the blood vessels of the so-called immunologically privileged sites, including the brain, testis, uterus, thyroid (Figure 3), ovary, and oviduct (data not shown). These results revealed that nepmucin expression shows extensive heterogeneity in different tissues and point to a possible role of nepmucin in immunological functions.

### Nepmucin/CD300LG expression is developmentally regulated during postnatal ontogeny

**Figure 4:**
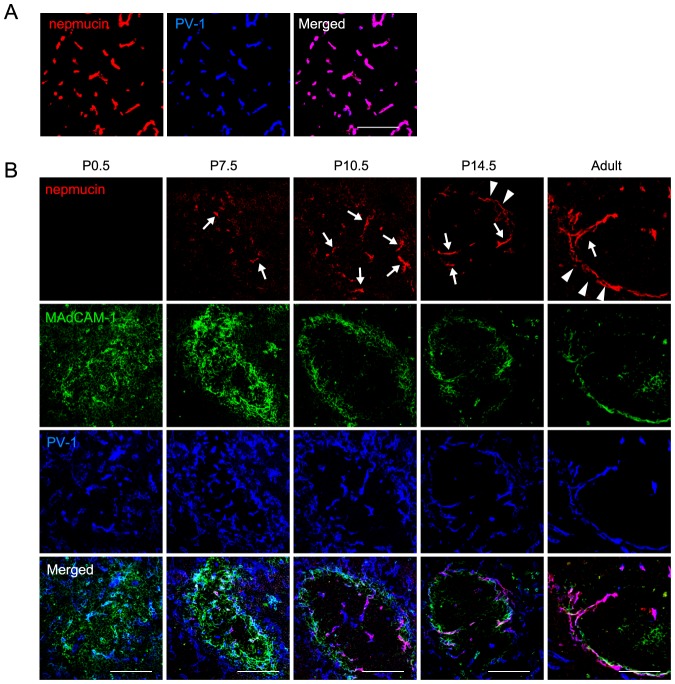
Nepmucin/CD300LG expression is developmentally regulated during postnatal ontogeny. The thymus was collected on P0.5 (A) and spleens were collected on P0.5, 7.5, 10.5, and 14.5, as well as from adult mice (B). Tissue sections were stained with an anti-MAdCAM-1 mAb (Alexa Fluor 488; green), anti-nepmucin mAb (Alexa Fluor 594; red), and anti-PV-1 mAb (Alexa Fluor 647; blue). In the spleen at P7.5-10.5, nepmucin was detected predominantly in microvessels leading to the marginal sinus (arrows). From P14.5 to adulthood, nepmucin was also found in the marginal sinus ECs (arrowheads). Scale bars, 100 µm.

We next examined nepmucin expression during postnatal development. In the thymus, nepmucin was detectable in newborn mice (Figure 4A). In the spleen, whereas no nepmucin expression was observed in the vasculature marked by PV-1 on postnatal day (P) 0.5 after birth (Figure 4B), by P7.5 after birth, punctate expression clearly appeared in the immature white pulp, where MAdCAM-1^+^ (a marker for lymphoid organizer cells/marginal reticular cells) [30] and PV-1^+^ cells aggregated. By P10.5, nepmucin was found in the PV-1^+^ vascular structure that extends from the marginal sinus toward the center of the white pulp, and by P14.5, it was strongly expressed in the marginal sinus surrounding the white pulp. These observations indicated that nepmucin expression is differentially regulated in organs during development, and that at least in the spleen, its postnatal expression is first weak and visible in immature vascular cells, but then becomes distinct in the ECs of particular vascular structures.

### Nepmucin/CD300LG expression is decreased during *in vitro* culture of ECs

**Figure 5:**
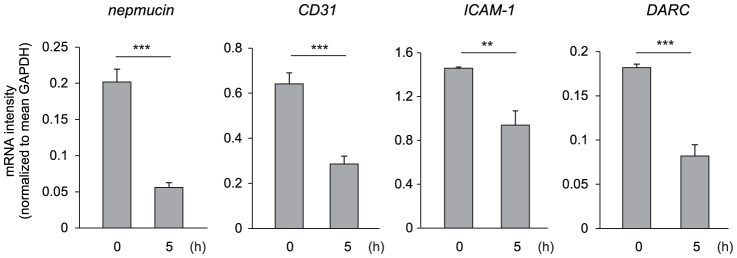
Nepmucin/CD300LG is rapidly down-regulated by *in vitro* culture of LN ECs. LN stromal cells that contain ECs were seeded on a collagen I-coated plate, and adherent cells were collected at time 0 or after 5-hours *in vitro* culture. The expression of *nepmucin, CD31, ICAM-1,* and *DARC,* was examined by quantitative PCR. Data represent the mean ± SD (n = 3 per group). **p<0.01, ***p<0.005.

The heterogeneous expression pattern of nepmucin in tissues leads us to speculate that nepmucin expression levels are regulated by local signal(s) from microenvironment. To test this hypothesis, we cultured freshly-prepared stromal cells that contain ECs of LNs *in vitro*, and monitored nepmucin expression by quantitative PCR. As shown in Figure 5, nepmucin expression was remarkably decreased at as early as 5 hours after initiation of cell culture. The expression of CD31, ICAM-1, and DARC (a high endothelial venule-associated promiscuous chemokine receptor [31] which was reported to undergo a rapid decrease during the EC culture [32]) also showed a significant decline, but a decrease in nepmucin expression was more prominent than that of other EC-related genes. These results are in line with the idea that nepmucin expression is under the control of the microenvironment at steady state.

### Nepmucin/CD300LG expression is down-regulated by inflammatory signals

**Figure 6:**
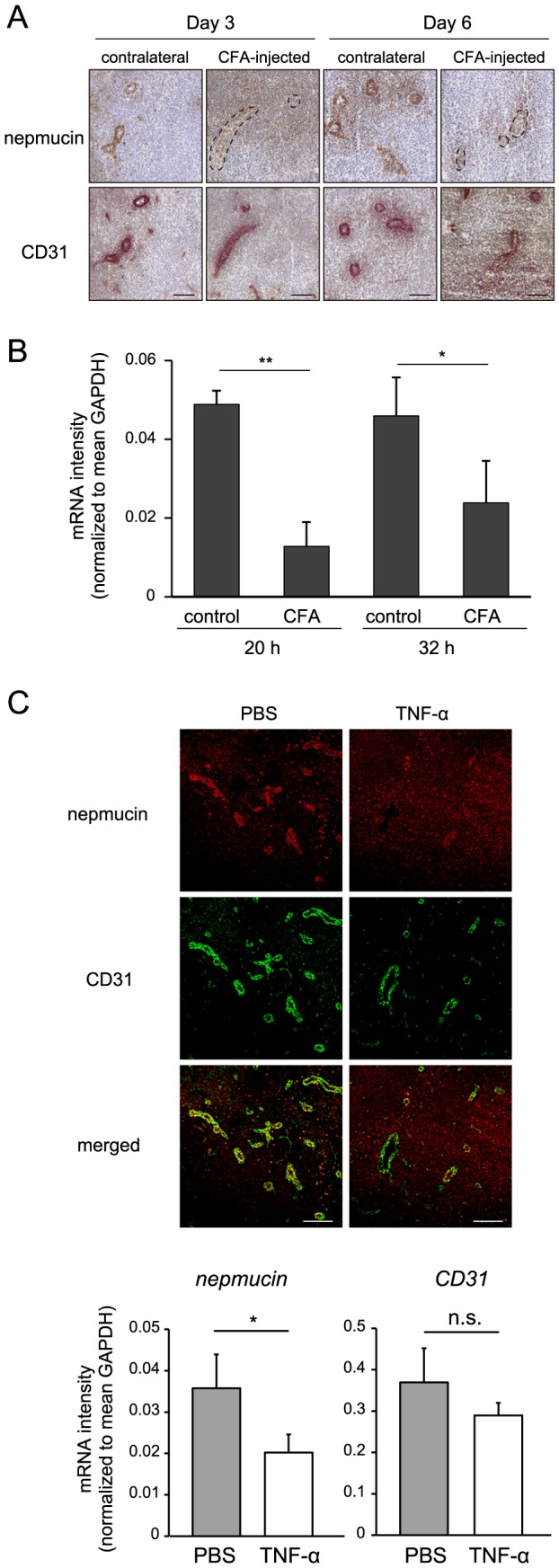
Nepmucin/CD300LG expression is down-regulated by inflammatory signals. (A, B) Draining LNs and contralateral LNs were harvested at the indicated time points after CFA was subcutaneously injected into the right footpad. In (A), the expressions of nepmucin and CD31 were analyzed by immunofluorescence staining. In (B), the nepmucin mRNA expression was assessed by quantitative PCR using primers that specifically recognize *nepmucin* gene. Data represent the mean ± SD (n = 3 per group) from three independent experiments. In (C), the nepmucin and CD31 expressions were examined by immunofluorescence staining (upper panel) and quantitative PCR (lower panel) of the draining LNs two days after the footpad injection of TNF-α (200 ng) or PBS. Data show representative images, n = 3-4 mice per group. *p<0.5, **p<0.01, ***p<0.005, n.s., not significant. Scale bars, 100 µm.

We next examined whether nepmucin expression is altered by locally produced signals at inflammatory sites. To this end, we subcutaneously injected CFA into mouse footpads and analyzed nepmucin expression in the draining LNs. As shown in Figure 6A, on days 3 and 6 after the injection, nepmucin expression was markedly decreased in the ECs of LNs draining the inflamed footpads compared to those of the contralateral LNs, whereas CD31 expression was apparently unaltered. A similar decrease in nepmucin expression was observed in the LNs draining the skin area where oxazolone was painted epicutanously (data not shown). As shown in Figure 6B, the level of nepmucin mRNA was significantly decreased in inflamed LNs, indicating that its expression is transcriptionally regulated.

Because TNF-α is a key player in inflammation, we examined its role in the regulation of nepmucin expression. As shown in Figure 6C, the subcutaneous injection of TNF-α but not PBS induced a marked decrease in nepmucin expression in the blood vessels of draining LNs, indicating that the constitutive expression of nepmucin is affected by acute inflammatory signals such as TNF-α.

### Nepmucin/CD300LG expression is decreased in tumors and tumor-draining LNs

**Figure 7:**
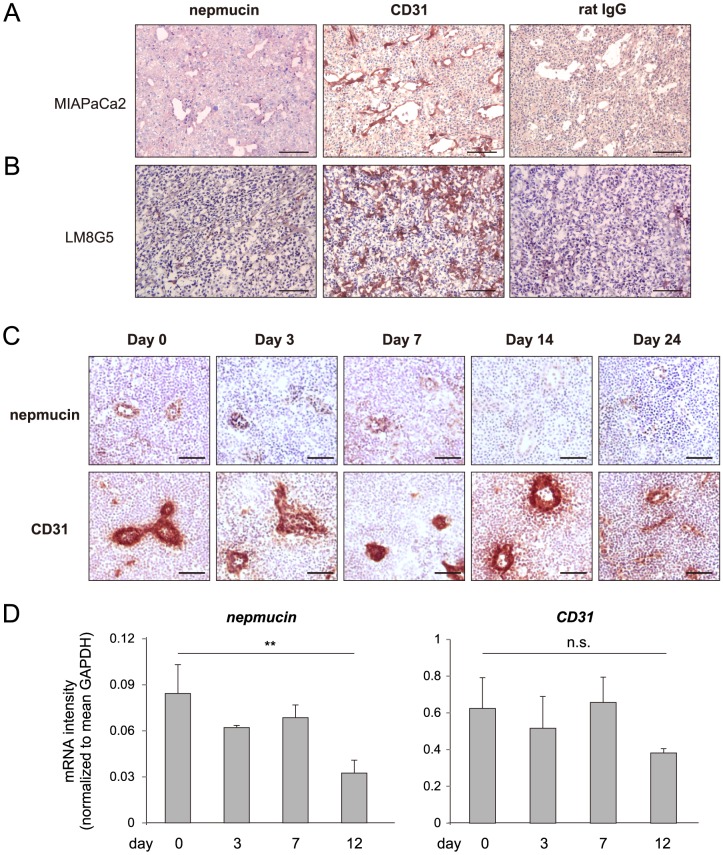
Nepmucin/CD300LG expression is decreased in tumors and tumor-draining lymph nodes. (A) Human pancreatic adenocarcinoma MIA PaCa-2 cells (1.2×10^7^ cells) were subcutaneously injected into the flank of SCID mice that had been treated with an anti-IL-2Rβ mAb. Eighteen days later, nepmucin expression was examined in the tumor tissues. (B) Nepmucin expression in liver metastatic tumors was examined after LM8G5 osteosarcoma cells (1×10^6^ cells) were intravenously injected into the ileocolic vein of C3H/HeN mice. (C, D) Axillary LNs were harvested from C57BL/6 mice that had been subcutaneously inoculated with 1×10^5^ B16-F10 melanoma cells. The nepmucin and CD31 expressions were examined by immunohistochemistry (C) and quantitative PCR (D) at the indicated time points after inoculation. Data show representative images, n = 3–4 mice per group. **p<0.01, n.s., not significant. Scale bars, 100 µm.

Because tumor blood vessels often have properties and phenotypes that differ from those of normal tissues, we examined nepmucin expression in tumors. As shown in Figure 7A, nepmucin was only marginally expressed in the vascular ECs of the pancreatic adenocarcinoma MIA PaCa-2, whereas CD31 was readily detected. A similar down-regulation of nepmucin was observed in the intrahepatic metastatic nodules of osteosarcoma LM8G5 (Figure 7B).

We next asked whether the nepmucin expression is also affected in tumor-related tissues. For this purpose, we injected B16-F10 melanoma cells subcutaneously into the flank of C57BL/6 mice, and performed immunohistochemistry of the draining LNs at different time points after the injection. As shown in Figure 7C, the nepmucin expression in the tumor-draining LNs decreased over time, and had almost disappeared by day 14 after the injection, which was confirmed by quantitative PCR analysis (Figure 7D). The down-regulation of nepmucin was not apparent in the non-tumor draining LNs (data not shown). In contrast, CD31 expression appeared unaltered until day 7 but declined only at day 12 after tumor implantation. Both LM5G8 and B16-F10 tumor cells produced undetectable levels of mouse TNF-α in the culture supernatant (< 25 pg/ml) as assessed by ELISA (data not shown). In addition, no apparent leukocyte infiltration was observed in the tumor tissue. Thus, tumor-derived TNF-α appears to play a minor role in the down-regulation of nepmucin expression. These observations are compatible with the idea that nepmucin expression is negatively regulated by local signal(s) produced by tumors, probably through a mechanism different from that observed in inflamed LNs.

### Nepmucin/CD300LG is induced in high endothelial venule-like blood vessels in chronically inflamed tissues and avidly binds inflammatory T cells

**Figure 8:**
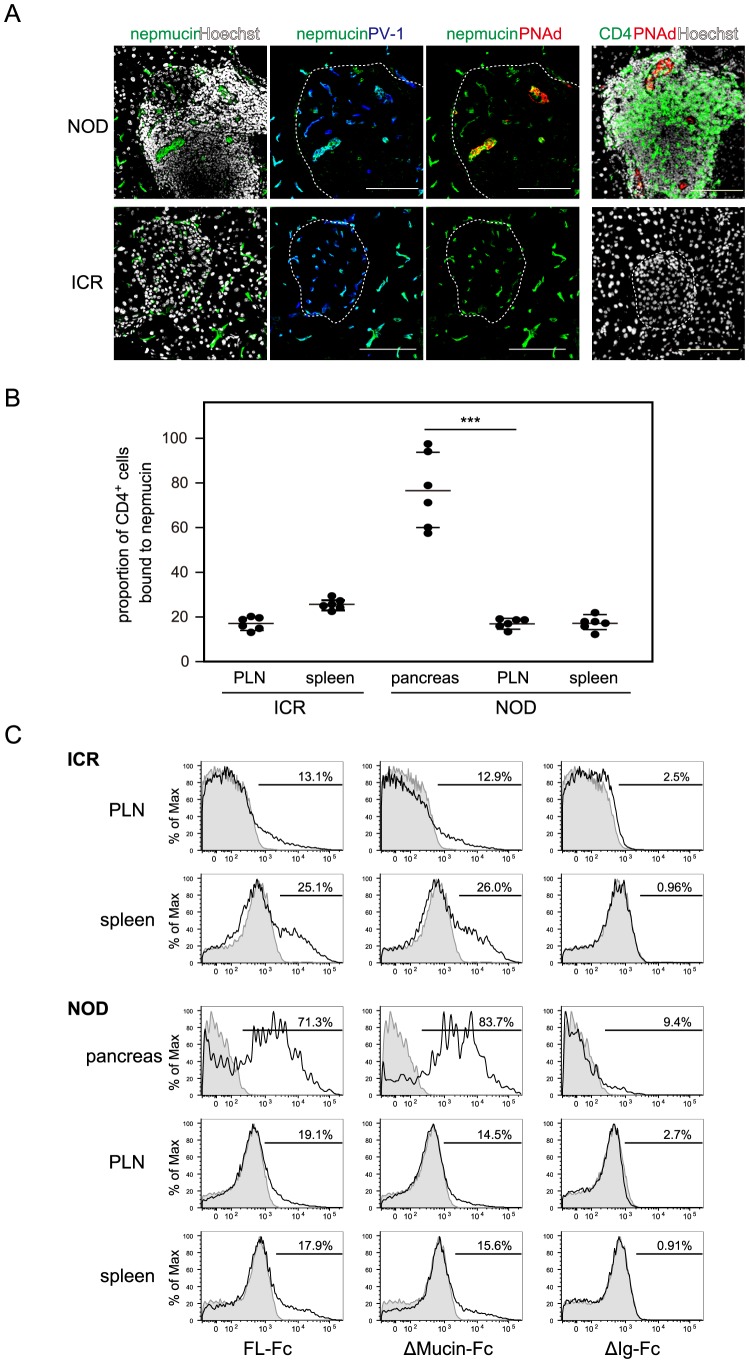
Nepmucin/CD300LG is expressed in NOD pancreas high endothelial venule-like vessels and avidly binds diabetes-associated CD4^+^ T cells. (A) The pancreas was collected from 13-week-old female NOD mice and ICR mice. Tissue sections were stained with anti-nepmucin mAb (green), anti-PV-1 mAb (blue), anti-PNAd mAb (red), and Hoechst 33432 (white). Alternatively, a frozen section was stained with anti-CD4 mAb (green), anti-PNAd mAb (red), and Hoechst 33432 (white). The Langerhans’s islets are delineated with dotted lines. Scale bars, 100 µm. (B, C) Lymphocytes prepared from the indicated tissues of NOD or ICR mice (15–19 weeks old) were incubated with admixed nepmucin-human IgG Fc chimeras and PE-conjugated F(ab’)_2_ anti-human IgG. The proportion of CD4^+^ T cells bound to full-length nepmucin (FL) is shown in (B), and representative histograms for the binding of nepmucin FL and its mutant (Δmucin; nepmucin mutant lacking the mucin-like domain, and ΔIg; nepmucin mutant lacking the Ig domain) are shown in (C). Data represent the mean ± SD (n = 6 mice per group) from two independent experiments. ***p<0.005.

Finally, we examined nepmucin expression in chronically inflamed tissues. It was previously reported that high endothelial venule-like vessels bearing L-selectin ligand activity appear in the chronically inflamed pancreas of NOD mice [19]. In agreement with this finding, we clearly observed high endothelial venule-like blood vessels that bound the MECA-79 mAb (and hence contained L-selectin-reactive PNAd sugar epitopes) in the pancreas of 4 out of 6 13-week-old female NOD mice. Within the NOD pancreatic islets, the nepmucin expression was particularly strong in PNAd^+^ PV-1^+^ blood vessels that morphologically resembled LN high endothelial venules, which contain tall and plump ECs, but neither the high endothelial venule-like blood vessel nor lymphocyte infiltration was observed in normal ICR mice from which NOD mice were initially derived (Figure 8A).

Because we previously reported that nepmucin binds to a proportion of lymphocytes and mediates their transendothelial migration via its Ig domain [23], we next examined the ability of nepmucin to bind lymphocytes that infiltrated chronically inflamed islets. As shown in Figure 8B, nepmucin bound to a substantially higher proportion of CD4^+^ T cells from the pancreas (76.6±16.8%) than to those from brachial LNs (16.9±2.0%) or the spleen (17.1±3.2%) of NOD mice. In age-matched control ICR mice, the nepmucin binding to CD4^+^ T cells from peripheral LNs or spleen was almost to the same as that observed in the NOD mice. The same tendency was observed for nepmucin binding to CD8^+^ T cells (data not shown).

The binding of nepmucin to pancreatic T cells was mediated by its Ig domain, given that full-length nepmucin (FL-Fc) or a mutant lacking the mucin domain (Δmucin-Fc) bound to lymphocytes, but a mutant lacking the Ig domain (ΔIg-Fc) did not (Figure 8C). These observations suggested that endothelial nepmucin can recognize inflammatory lymphocytes and support the idea that nepmucin is involved in the recruitment of inflammatory lymphocytes in chronically inflamed tissues.

## Discussion

In this study, we showed that the Ig domain-containing sialomucin nepmucin/CD300LG exhibited specific expression patterns in the microvascular ECs of different tissues. Nepmucin/CD300LG was expressed in the microvessels, including small arterioles, venules, and capillaries, of many but not all tissues. For instance, it was expressed in the microvascular ECs of the splenic white pulp, thymic cortex, and LNs, but only weakly in the splenic red pulp or thymic medulla, and it was not detectable in immunologically privileged sites, including the brain, testis, and uterus.

This heterogeneous expression pattern is unique in terms of its selectivity for both the vascular EC type and the organ/tissue type. This pattern differs from those of other endothelial transmembrane proteins. For example, ICAM-1 and VCAM-1 are constitutively expressed in venular ECs and are up-regulated in inflamed tissues, whereas CD31 is widely expressed in various ECs in most tissues [6]. P-selectin is constitutively expressed in venular ECs in various tissues with the exception of brain, and absent from the ECs of capillaries and arterioles [33]. PV-1 is expressed in medium-sized vessels of the lung and intestinal villi, but not in the brain, heart, or kidney glomeruli [8], [9]. Thus, the nepmucin/CD300LG expression pattern is quite different from those of other EC molecules, suggesting that the gene expression for nepmucin/CD300LG is uniquely regulated.

A number of genes that regulate arterial and venous differentiation have been described, although to our knowledge, no gene that specifically discriminates small arterioles/venules from relatively large vessels has been reported. Our findings raise the possibility that the differentiation or maturation of small arterioles/venules is directed by specialized programming, and that small vessels exhibit intrinsically specific gene characteristics that are distinct from those of large arterioles/venules.

It is likely that the nepmucin/CD300LG expression is induced by microenvironment-derived signals, such as shear stress, oxygen partial pressure, nutrients, and so on. The shear stress in the capillary is estimated to be greater than that in major vessels [34], and to date, several shear stress-responsive elements (SSREs) have been identified in the promoter region of certain endothelial genes. For instance, GAGACC is found in the promoters of genes encoding platelet-derived growth factor B (PDGFB) [35], CD31 [36], and nitric oxide synthase 3 (eNOS) [37], and TGACTCC (TPA response element) is found in the CCL2/MCP-1 promoter [38]. Our preliminary analysis *in silico* revealed a TGACTCC sequence in the promoter region of the nepmucin/CD300LG gene (4.7-kb upstream of the gene) (data not shown). Further study is needed to determine whether nepmucin/CD300LG expression is regulated by specific signals from the vascular bed milieu.

Different vascular beds exhibit extensive internal regional variations in gene expression in vivo [5], [39], [40], and a variety of gene promoters have been described that can affect gene expression patterns in ECs [41]. For human von Willebrand factor (vWF), a DNA sequence between -843 and -620 is necessary for its expression in capillaries, whereas the first intron is required for its expression in large blood vessels of the heart and skeletal muscle as well as in capillaries [42], indicating that a combination of particular DNA modules regulates the vascular bed-specific expression of vWF. Likewise, nepmucin/CD300LG expression may be controlled by its promoter and/or regulatory elements in a tissue-intrinsic manner. Given that the transcriptional regulation of endogenous genes is rarely mediated by a single promoter, the unique expression of nepmucin/CD300LG is likely to be regulated by both extrinsic signals dependent on the local microenvironment (shear stress, etc.) and signals intrinsic to the programming of individual tissues.

An interesting question is the biological rationale for the absence of nepmucin/CD300LG expression from immunologically privileged sites such as the brain, testis, and uterus, and also the tumor-related tissues. We previously reported that nepmucin/CD300LG mediates lymphocyte transendothelial migration across ECs, at least in vitro [23]. In addition, nepmucin bound to CD44^+^ effector/memory cells more strongly than to naïve lymphocytes in secondary lymphoid tissues under homeostatic conditions (unpublished data). Thus, it is tempting to speculate that the absence of nepmucin/CD300LG expression contributes to the lack of accessibility of immune effector cells to these tissues, conferring on them their immune-privileged status. The down-regulation of nepmucin/CD300LG expression in tumor blood vessels might also play a role in making immune effector cells inaccessible to tumor tissues.

The nepmucin/CD300LG expression was rapidly down-regulated by inflammatory signals such as TNF-α. As described above, nepmucin expression was also decreased in the ECs of tumors and tumor-draining LNs, leading us to speculate that tumors secrete certain soluble factors that negatively regulate nepmucin expression, and that these tumor-derived factors are transported to the draining LNs via lymph. Tumors are reported to produce secretary factors that can regulate the EC phenotype. For instance, tumor interstitial fluid from colon adenocarcinoma increases the cell-surface expression levels of ICAM-1 and VCAM-1 in HUVEC cells, and tumor-derived angiogenic factors such as VEGF modulate the expression of such adhesion molecules [16]. The conditioned medium from Lewis carcinoma or B16-F1 melanoma cells up-regulates the promoter activity and mRNA expression of Flt-1 [43]. Thus, it is likely that tumor-associated factors modulate nepmucin/CD300LG expression, although further study is required to identify the factors.

Nepmucin/CD300LG was strongly expressed in high endothelial venule-like vessels in the pancreatic islets of NOD mice and that it bound more strongly to the T cells in the pancreas than to those in the LNs or spleen of these mice. Given that nepmucin/CD300LG strongly binds to effector/memory cells as described above, the presence of a high proportion of nepmucin-binding lymphocytes supports the notion that these T cells are activated. In line with this idea, a previous study showed that the islet-infiltrating lymphocytes are mainly of the L-selectin^−/low^ LFA-1^high^ CD44^high^ integrin α4β7^high^, activated phenotype [20]. Our findings raise the possibility that nepmucin binding serves as a good marker for the inflammatory T cells infiltrating the islets during diabetes development in NOD mice.

An obvious question is the function of the nepmucin/CD300LG expressed by the high endothelial venule-like blood vessels of the Langerhans’ islets in NOD mice. We previously reported that nepmucin/CD300LG mediates L-selectin-dependent lymphocyte rolling via its mucin-like domain upon the appropriate sugar chain modification of this domain, and regulates lymphocyte binding as well as lymphocyte transendothelial migration across ECs, at least in vitro [21], [23]. One possibility is that nepmucin expressed in the pancreatic ECs serves as an L-selectin ligand to promote lymphocyte rolling upon the appropriate oligosaccharide modification of its mucin-like domain. Another possibility, not mutually exclusive, is that nepmucin facilitates lymphocyte infiltration into islets through interactions with non-L-selectin ligand(s) on the lymphocytes. Further investigation is required to clarify the role of nepmucin/CD300LG in lymphocyte infiltration into pancreatic islets.

In conclusion, our results demonstrated that nepmucin/CD300LG is preferentially expressed in microvessels, with specific expression patterns in tissues, reflecting the heterogeneity of ECs. Nepmucin expression is rapidly down-regulated by acute inflammatory or tumor-associated signals. By contrast, it is induced in chronically inflamed pancreatic ECs and avidly binds to activated T cells infiltrating the pancreatic islets. The functional role of human nepmucin/CD300LG merits further detailed investigation.
